# Balanced steady-state free precession phase contrast at 0.55T applied to aortic flow

**DOI:** 10.1016/j.jocmr.2024.101098

**Published:** 2024-09-13

**Authors:** Jie Xiang, Rajiv Ramasawmy, Felicia Seemann, Dana C. Peters, Adrienne E. Campbell-Washburn

**Affiliations:** a Department of Biomedical Engineering, Yale University, New Haven, Connecticut, USA; b Cardiovascular Branch, Division of Intramural Research, National Heart, Lung, and Blood Institute, National Institutes of Health, Bethesda, Maryland, USA; c Department of Radiology and Biomedical Imaging, Yale University, New Haven, Connecticut, USA

**Keywords:** Aortic flow, 0.55T MRI, CMR, Phase contrast, bSSFP

## Abstract

**Background:**

There is a growing interest in the development and application of mid-field (0.55T) for cardiovascular magnetic resonance (CMR), including flow imaging. However, aortic flow imaging at 0.55T has limited signal-to-noise ratio (SNR), especially in diastolic phases where there is reduced inflow-driven contrast for spoiled gradient recalled echo (GRE) sequences. The low SNR can limit the accuracy of flow and regurgitant fraction measurements.

**Methods:**

In this work, we developed a two-dimensional phase contrast (PC) acquisition with balanced steady-state free precession (bSSFP), termed PC-SSFP, for flow imaging and quantification at 0.55T. This PC-SSFP approach precisely nulls the zeroth and first gradient moments at both the echo time (TE) and repetition time, except for the flow-encoded acquisition, for which the first gradient moment at the TE is determined by the velocity encoding. Our proposed sequence was tested in both phantoms and in healthy volunteers (n = 11), to measure aortic flow. In volunteers, both a breath-hold (bh) and a free-breathing (fb) protocol, with averaging to increase SNR, were obtained. Total flow, peak flow, cardiac output, and SNR were compared for PC-SSFP and PC-GRE. Stroke volumes were also measured and compared to planimetry method.

**Results:**

In a phantom, SNR was significantly higher using PC-SSFP compared to PC-GRE (25.5 ± 9.6 vs 8.2 ± 2.9), and the velocity measurements agreed well (R = 1.00). In healthy subjects, for both bh and fb protocols, PC-SSFP measured accurate peak flow (fb: R = 0.99, bh: R = 0.96) and cardiac output (fb: R = 0.98, bh: R = 0.88), compared to PC-GRE, accurate stroke volume (fb: R = 0.94, bh: R = 0.97), compared to planimetry measurement, and offered constant high SNR (fb: 28 ± 9 vs 18 ± 6, bh: 24 ± 7 vs 11 ± 3) over the cardiac cycle in 11 subjects.

**Conclusion:**

PC-SSFP is a more reliable evaluation tool for aortic flow quantification, when compared to the conventional PC-GRE method at 0.55T, providing higher SNR, and thus potentially more accurate flows.

## Graphical abstract

**Figure:**
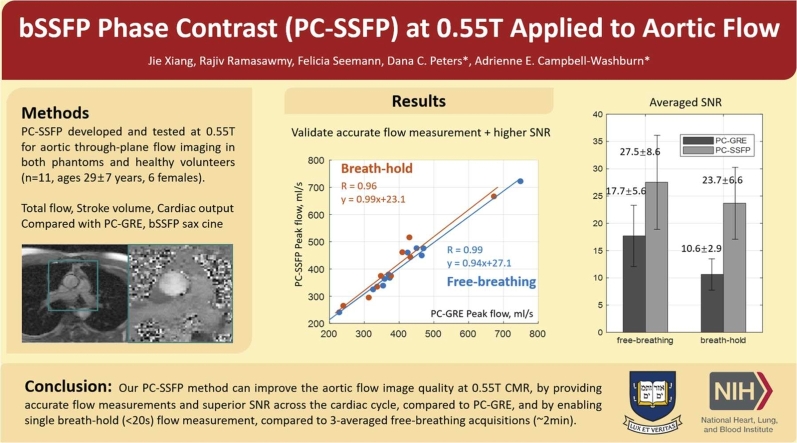

## 1 Background

Phase contrast (PC) is an essential tool for cardiovascular magnetic resonance (CMR) [1], [2], [3] to measure flow volumes and velocities of the great vessels, including the aorta and pulmonary artery. These measurements are used clinically for evaluating stroke volume, cardiac output (CO), regurgitation, stenotic flow, and even pulse wave velocity.

Mid-field magnetic resonance imaging systems (0.55T) have recently been demonstrated for CMR and may improve patient access to CMR and offer new clinical opportunities for both structural and functional assessment [4], [5]. However, the reduced signal-to-noise ratio (SNR) at 0.55T is problematic for certain CMR sequences, especially PC which is gradient recalled echo (GRE) based. PC provides low (but acceptable) SNR even at conventional field strengths, especially in diastole when there is no signal enhancement due to inflow contrast. This problem can be alleviated by modifying scan parameters (e.g., receiver bandwidth, and averages), with the compromise of longer scan time and increased risk of motion-related artifacts if scans are acquired during free-breathing (fb).

Balanced steady-state free precession (bSSFP) has intrinsically high blood SNR and preferable myocardium-blood contrast [6]. Previous studies have successfully combined PC with bSSFP for flow imaging, termed PC-SSFP [7], [8], [9], [10], [11]. Limitations of PC-SSFP include prolonged repetition time (TR), which can lead to undesirable banding at conventional field strength, and more challenging flow quantification due to bSSFP phase behavior. Recently, PC-SSFP has been investigated at 0.55T [12], [13]. McGrath et al. [12] used a bSSFP pulse sequence with inversion of the slice-select gradient to generate the negative bipolar, as previously developed [7]. They found 2.9-fold increased SNR vs PC-GRE at 0.55T, and reasonable flow accuracy, except overestimation at high velocities.

We previously presented an in-plane PC-SSFP method for all-in-one diastolic dysfunction evaluation at 3T [14] with a different approach, by designing M_0_ and M_1_ gradient moments at both the TR and echo time (TE) to reduce velocity errors. This tactic is similar to other multi-echo and a single-echo PC-SSFP methods [9], [10], [11], but with a shorter TR (∼4 ms at 3T). Compared to other approaches, uncoupling the velocity encoding from slice-selection process yields more flexibility and high accuracy.

Here, we have translated our in-plane PC-SSFP method for 0.55T CMR and through-plane flow. We hypothesized that our method would yield similar flow measurements, but higher SNR compared to PC-GRE, enabling flow imaging at 0.55T in one breath-hold (bh) without the need for averaging. We assess through-plane aortic flow measurement in healthy volunteers and confirm quantification in a flow phantom.

## 2 Methods

### 2.1 PC-SSFP sequence

**Fig. 1:**
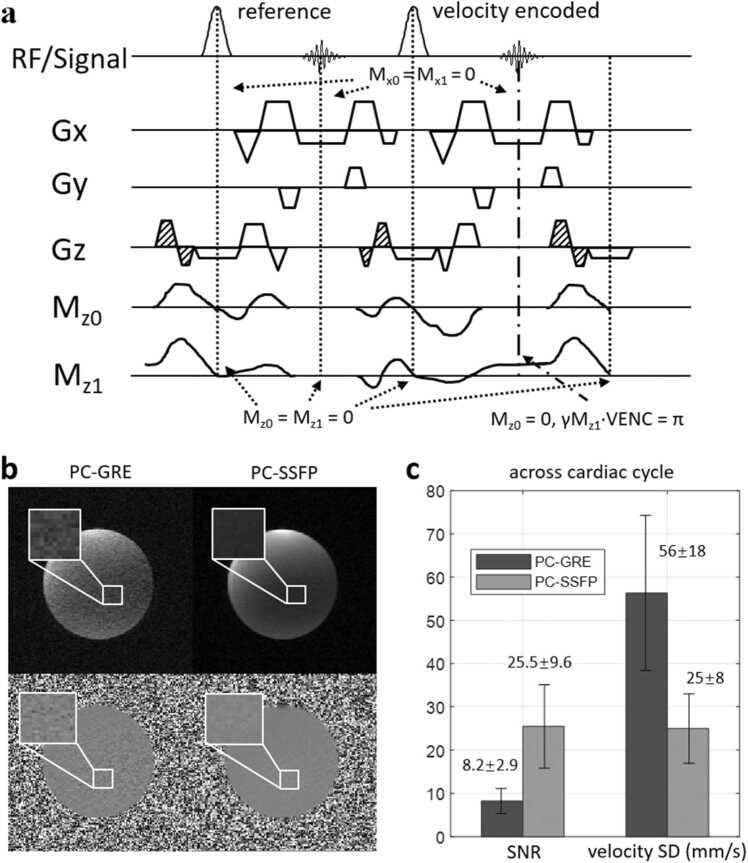
(a) Diagram of the proposed through-plane PC-SSFP sequence using velocity encoding in the slice-selection direction (Gz). Zeroth moment (M_z0_) and first moment (M_z1_) are all nulled at center of the readout and end of each TR, except that M_z1_ for the flow-encoded acquisition is determined by the VENC. (b) Comparison of magnitude and phase images between PC-GRE and PC-SSFP in a static phantom. (c) PC-SSFP showed significantly improved SNR (*p* < 0.001) and lower phase SD (*p* < 0.001) compared to PC- GRE (studied over 30 cardiac frames). *PC-SSFP* phase contrast acquisition with balanced steady-state free precession, *TR* repetition time, *VENC* velocity encoding, *PC-GRE* phase contrast gradient recalled echo, *SNR* signal-to-noise ratio, *SD* standard deviation, *RF* radiofrequency

Fig. 1a presents the sequence diagram for the proposed through-plane PC-SSFP acquisition at 0.55T. An α/2 preparation scheme was used at the beginning of the acquisition to bring the magnetization into steady state. In each TR, zeroth moment (M_z0_) and first moment (M_z1_) of the slice-selection gradients, Gz, were all nulled at the TE (center of the readout), and at the TR, except that M_z1_ at the TE for the flow-encoded acquisition is determined by the chosen velocity encoding (VENC). Importantly, bipolar gradients were added, and merged with slice-selection prephasing lobe for shorter TR (gradients filled with diagonal stripes in Fig. 1a), to set the M_z0_ and M_z1_ at the end of the TR to zero, similar to our in-plane approach for 3T [14]. Since flow was not encoded in the readout direction, Gx gradients were always flow compensated (M_x0_ and M_x1_ were nulled at all TE and TR for both reference and VENC acquisitions). First moment nulling at the TE is standard for PC to reduce signal loss from flow dephasing. Gy was only zeroth moment nulled. We did not design the sequence to null the moments of higher order. For this PC-SSFP method, just as for PC-GRE (but different than many PC-SSFP methods), the reference and VENC acquisitions were acquired in alternate TRs, since the steady state was not disturbed by phase-accumulations. For comparison, we also acquired reference and VENC TR separately, given that first moment was nulled at any TR.

### 2.2 Phantom studies

All imaging experiments were conducted with a 0.55T scanner (prototype MAGNETOM Aera, Siemens Healthcare, Erlangen, Germany) [15] with phantoms and subjects approximately at isocenter. This system was created by ramping down a clinical 1.5T system and maintaining the gradient specifications (maximum gradient 43 mT/m and slew rate 180 T/m/s). A 12-channel prototype cardiac and lung coil was used for signal reception (NeoCoil LLC, Pewaukee, Wisconsin, USA) [16]. A static phantom was imaged with PC-GRE and PC-SSFP to compare SNR and velocity standard deviation (SD). To validate PC-SSFP’s through-plane flow quantification, a tap-water-filled flow phantom was then studied. A tube connected to a peristaltic pump motor (Omegaflex, Stamford, Connecticut, USA), which generated water flow with constant mean velocities set to range from 0 to 100 cm/s, was wrapped around a cylindrical static phantom. The mean velocities at seven different pump power settings were measured by PC-GRE and PC-SSFP. The simulated RR was set to 1000 ms.

PC-SSFP used the following scan parameters: TR/TE/θ = 5.6 ms/2.5 ms/60°, FOV = 270× 360 mm, base resolution = 192, slice thickness = 8 mm, VENC = 150 cm/s, 66%(2.2ms/3.3ms) asymmetric-echo, electrocardiogram (ECG)-retrospectively gated, two views per segment, 30 cardiac phases, bandwidth = 300 Hz/pixel, temporal resolution 23 ms, one signal average, and scan time ∼40 s. Our previously published 3T protocol was adapted to 0.55T to use a lower bandwidth and thus longer TR, for increased SNR, without penalty related to bSSFP banding artifacts [12]. PC-GRE used similar parameters, previously optimized [15], except TR/TE/θ = 7 ms/4.3 ms/15°, temporal resolution 28 ms, and had matched spatial resolution and approximately matched scan time ∼40 s.

### 2.3 Healthy volunteer studies

All subjects provided written informed consent, and the study was approved by the Institution’s institutional review board (ClinicalTrials.gov identifier NCT03331380). Eleven healthy subjects (ages 29 ± 7 years, 6 females) were imaged in an axial slice for through-plane flow measurements of the ascending aorta.

We used identical scan parameters as described for phantom measurements, except for signal averaging. Four PC acquisitions were performed: breath-held PC-GRE and PC-SSFP with four views per segment (temporal resolution = 56 ms and 44.8 ms, respectively, scan time ∼20 s) and one signal average, and fb PC-GRE and PC-SSFP with three averages (two views per segment as in phantom study, scan time ∼2 min). The fb scans were reconstructed online with averaging across the respiratory cycle, per the previously established interventional CMR protocol using PC-GRE [17]. This protocol was chosen to test PC-SSFP against a fb PC-GRE previously configured to capture CO averaged across the respiratory cycle, and to demonstrate the feasibility of PC-SSFP to quantify aortic flow in a single bh.

Additionally, a fb short-axis stack of bSSFP cines covering the left ventricle (LV) was acquired and reconstructed using the Gadgetron [18], as a second independent measurement of stroke volume. The scan parameters were TR/TE/θ = 1.35 ms/3.3 ms/80°, FOV = 365 × 274 mm, in-plane resolution = 1.9 × 1.9 mm, slice thickness = 8 mm, slice gap = 2 mm.

### 2.4 Image analysis

**Fig. 2:**
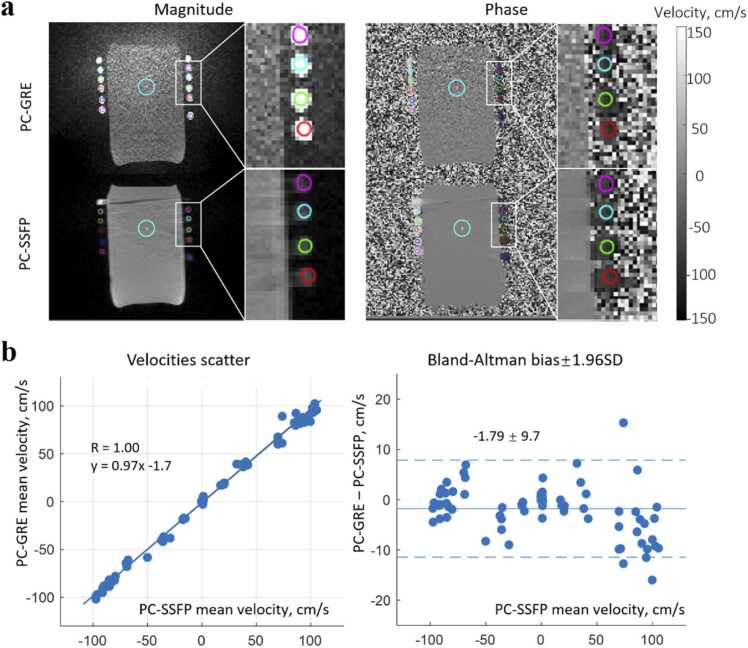
Flow phantom experiment using the through-plane PC-SSFP sequence. (a) Seven different pumping rates between 0 and 100 cm/s were investigated, with velocity measured in 11 ROIs. Inward blurring in the readout direction was observed between the tube and cylindrical phantom possibly due to partial volume and out-of-slice contribution of the flowing spins. (b) Scatter plot and Bland-Altman analysis. Mean flow velocities measured by PC-GRE and PC-SSFP showed excellent correlation and agreement. Identical coils were used for both methods. *PC-SSFP* phase contrast acquisition with balanced steady-state free precession, *PC-GRE* phase contrast gradient recalled echo, *ROI* region of interest

All images for flow analysis were from automatic online reconstruction. Images were then processed and evaluated in Matlab (Matlab 2019b; MathWorks, Natick, Massachusetts) using Segment software 3.2 R8836 (Medviso, Lund, Sweden) [19]. Automatic linear eddy current compensation was applied first for all PC sequences in the Segment software. In the phantoms, the velocity SD at each timeframe was calculated within regions of interest (ROIs) drawn on static phantom, where ideally phase should be constantly zero. The SNR at each pixel was calculated using the mean intensity over 30 acquired cardiac phases divided by the SD across the 30 phases. In the constant flow phantom, the mean velocity within each ROI was also averaged over the 30 phases for higher SNR and more robust comparison of velocities by PC-GRE and PC-SSFP. Ten ROIs with similar size as the tube cross-section and one larger ROI representing the static phantom at the center were studied for each flow rate (Fig. 2a).

**Fig. 3:**
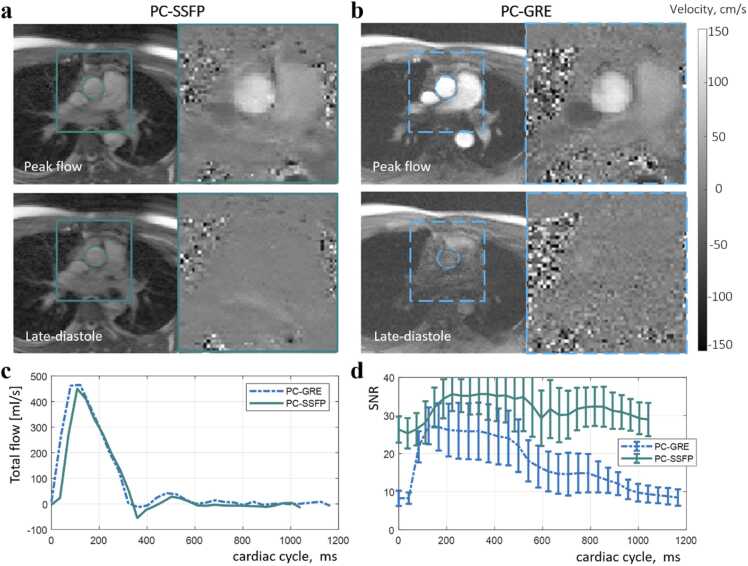
Free-breathing PC-SSFP (a) vs PC-GRE (b) in a representative healthy subject (movies uploaded as Supplemental Material S4). PC-SSFP maintained accurate flow vs PC-GRE (c) and constant high SNR (d). There was a slight shift between the two flow curves possibly due to the frame interpolation during reconstruction, considering the different heart rates in this case (1041 vs 1165 ms). Note the image and phase noise provided by PC-GRE were high even with three averages, which was visually evident during diastole. In contrast PC-SSFP, SNR was less phasic and SNR was nearly tripled in late diastole vs PC-GRE. *PC-SSFP* phase contrast acquisition with balanced steady-state free precession, *PC-GRE* phase contrast gradient recalled echo, *SNR* signal-to-noise ratio

**Fig. 4:**
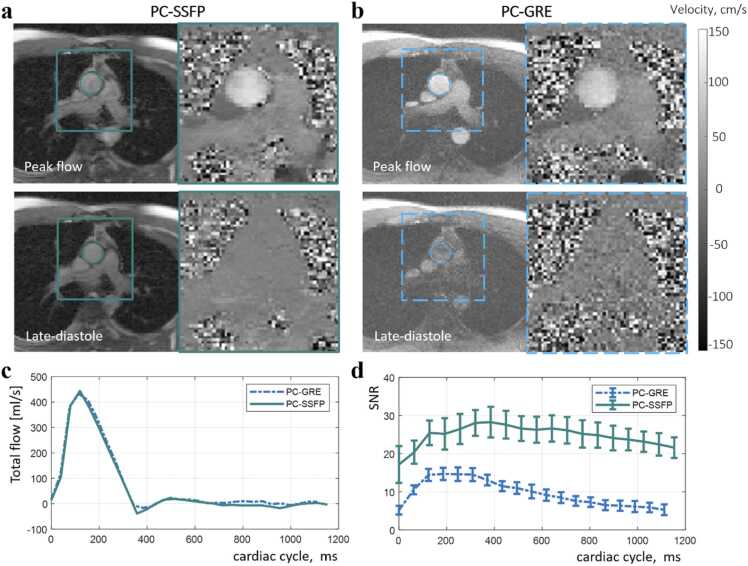
Breath-hold PC-SSFP (a) vs PC-GRE (b) in a representative healthy subject (movies uploaded as Supplemental Material S5). PC-SSFP provided similar flow vs PC-GRE (c) and was able to capture more negative flow in dicrotic notch likely due to improved SNR (d). Inflow enhancement was found in both PC-GRE and PC-SSFP but PC-SSFP maintained adequate high SNR, thus expected high velocity-to-noise ratio, across the whole cardiac cycle, without averaging. *PC-SSFP* phase contrast acquisition with balanced steady-state free precession, *PC-GRE* phase contrast gradient recalled echo, *SNR* signal-to-noise ratio

In aortic flow curves of healthy subjects, peak flow values, CO, and stroke volumes were measured using the PC-SSFP and PC-GRE scans. Aortic ROIs were drawn as shown in Figs. 3 and 4. These ROIs were contoured on one frame and automatically propagated to the other frames to track vessel motion, with manual correction if needed. This correction was performed blinded to the final stroke volumes to avoid reader bias in segmentation. The measurements for each scan were compared, to determine the agreement of PC-SSFP vs PC-GRE using the fb and bh protocols. The stroke volumes by planimetry from cine imaging were measured as LV cavity volume difference between end-diastole and end-systole. The LV segmentation of each short-axis slice was performed manually in the Segment software.

As velocity-to-noise ratios (VNR) are proportional to SNR, i.e. SNR * velocity/VENC [20], we compared the SNRs over the cardiac cycle for PC-SSFP and PC-GRE. To estimate SNR on a per pixel basis, PC images were also reconstructed in SNR units using a pseudo-replica method [21], [22]. The mean SNR within the aortic ROIs was measured.

### 2.5 Statistical analysis

Time-averaged SNR and velocity SD for phantom and in vivo experiments were compared using a paired t-test in Matlab. Peak flow values, stroke volumes, and CO were investigated using linear regression, Pearson correlation coefficients and Bland-Altman analysis were used to compare PC-SSFP and PC-GRE. The correlations between bh and fb acquisitions, PC, and planimetry stroke volumes were also studied. An R > 0.6 was regarded as strong correlation. A *p*-value <0.05 was considered statistically significant.

## 3 Results

### 3.1 Phantom studies

Fig. 1b shows the magnitude (top) and phase (bottom) images of PC-GRE (left) and PC-SSFP (right) in the static phantom experiment. PC-GRE demonstrated visually higher noise in both intensity and velocity measurements. Statistically, SNR was significantly improved using PC-SSFP compared to PC-GRE (25.5 ± 9.6 vs 8.2 ± 2.9, *p* < 0.001, three-fold increase). The velocity SD (mm/s) was much lower with PC-SSFP (25.0 ± 8.0 vs 56.3 ± 18.0, *p* < 0.001), indicating improved precision.

In the flow phantom study (images in Fig. 2a), higher SNR and lower phase SD could be observed visually in PC-SSFP again, especially in regions with less coil sensitivity. Mean flow velocities calculated with PC-SSFP and PC-GRE showed excellent agreement across all flow velocities ranging from 0 to 100 cm/s (Fig. 2b, R = 1.0; bias ± 1.96 SD = −1.79 ± 9.7 cm/s). It is worth noting that the accurate measurements obtained with PC-GRE were aided by averaging over the cross-section, which mitigates noise. A flow profile across the tube cross-section and a pixel-wise comparison within the large ROI at the center of FOV (Supplemental Fig. S1) depicted the SNR improvement using PC-SSFP, compared to PC-GRE.

### 3.2 In vivo studies

In our 11 subjects, all PC-SSFP scans provided usable images at 0.55T. Some subjects showed aliasing in the first several cardiac phases (≤3 out of 30 phases), during the approach to steady state (N = 4), minor banding artifacts on chest wall (N = 3) and flow-related transient signal during peak flow (N = 1) that had limited effect on aortic flow measurement. Motion-induced artifacts were recognized in N = 3 subjects, in both PC-SSFP and PC-GRE, but only fb acquisitions. Supplemental Fig. S2 summarizes image magnitude and phase examples for these artifacts. Supplemental Fig. S3 shows PC-SSFP artifacts when acquiring reference and VENC TRs separately (as opposed to interleaved). These acquisitions were more sensitive to motion and did not necessarily provide the same number of reference and VENC TRs using ECG-retro gating, leading to more complexity in reconstruction. Therefore, we focused on analyzing the interleaved acquisitions in this work.

### 3.3 PC-SSFP vs PC-GRE

Figs. 3 and 4 compare PC-SSFP and PC-GRE at 0.55T in a representative subject. The flow curves of the ascending aorta agreed during both fb (Fig. 3c) and bh (Fig. 4c) acquisitions, except that PC-SSFP measured more negative flow (backward volume fb: −6.3 mL vs −1.4 mL, bh: −5.4 mL vs −1.6 mL). In this subject, improved SNR across the cardiac cycle with PC-SSFP vs PC-GRE was evident (fb: 31.8 ± 3.0 vs 17.1 ± 6.8), and PC-GRE exhibited low SNR in late diastolic phases even with three averages (PC-SSFP 30.9 ± 1.2 vs PC-GRE 10.1 ± 2.7). For the bh acquisitions with one average, PC-SSFP still provided consistently high SNR (bh: 24.7 ± 2.7 vs 9.6 ± 3.3) thus high VNR at mid-field strength. The movies of aortic phase contrast, including pseudo-replica SNR-unit reconstructions, are provided as Supplemental Material S4 and S5.

**Fig. 5:**
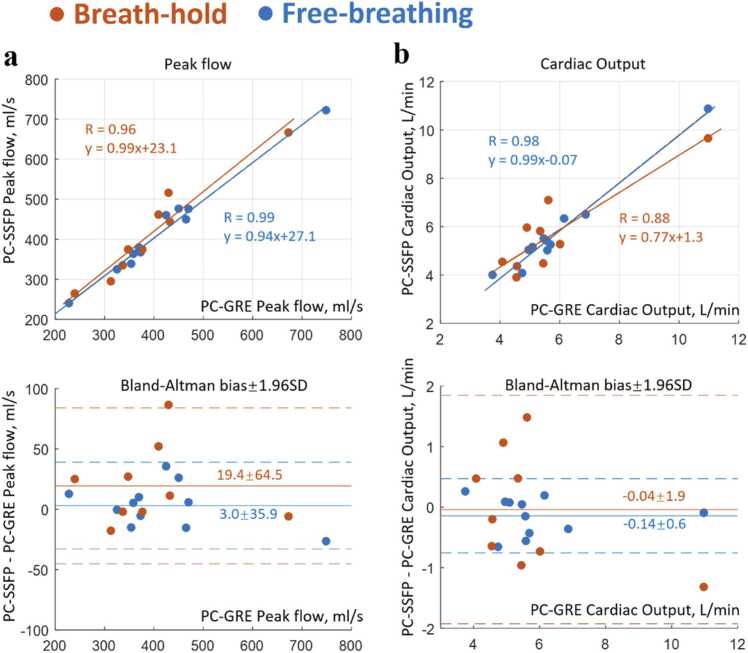
Eleven subjects imaged under free-breathing and breath-hold. Our proposed PC-SSFP (a) measured accurate peak flow compared to standard PC-GRE, (b) yielded similar cardiac output, though breath-hold PC-SSFP and PC-GRE had less strong correlation regarding cardiac output. *PC-SSFP* phase contrast acquisition with balanced steady-state free precession, *PC-GRE* phase contrast gradient recalled echo, *SD* standard deviation

Over the 11 subjects (Fig. 5) imaged during free-breathing, PC-SSFP measured similar peak flows, PF, to PC-GRE (linear regression PF_PC-SSFP_ = 0.94PF_PC-GRE_ + 27.1 mL/s, R = 0.99; bias ± 1.96 SD = 3.0 ± 35.9 mL/s) and CO (CO_PC-SSFP_ = 0.99CO_PC-GRE_ − 0.07, R = 0.98; −0.14 ± 0.6 L/min). For bh acquisitions, peak flow measurements also showed excellent correlation (PF_PC-SSFP_ = 0.99PF_PC-GRE_ + 23.1 mL/s, R = 0.96; 19.4 ± 64.5 mL/s). COs measured during bh showed less strong agreement (CO_PC-SSFP_ = 0.77CO_PC-GRE_ + 1.3 L/min, R = 0.88; −0.04 ± 1.9 L/min) likely due to the low SNR of PC-GRE in diastolic phases.

### 3.4 Breath-hold vs free-breathing

**Fig. 6:**
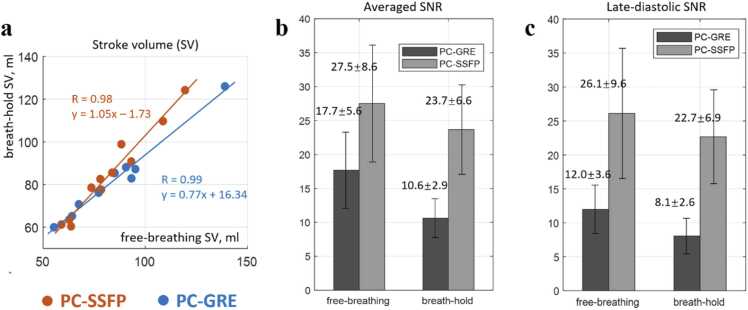
(a) The stroke volumes from breath-hold and free-breathing PC acquisitions correlated well for both PC-GRE and PC-SSFP. PC-SSFP had a slope closer to unity and smaller bias. (b) Histogram of averaged SNR across the whole cardiac cycle for the four PC acquisitions evidenced much improved images and flow measurements using our proposed PC-SSFP method in both free-breathing and breath-hold. (c) The PC-SSFP SNR was less flow-dependent while PC-GRE showed very limited SNR in late diastolic phases even with three averages. *PC-SSFP* phase contrast acquisition with balanced steady-state free precession, *PC-GRE* phase contrast gradient recalled echo, *SNR* signal-to-noise ratio

Mean and SD for bh lengths were PC-SSFP 19.5 ± 3.1 s vs PC-GRE 19.2 ± 3.0 s across 11 healthy volunteers. When comparing the stroke volumes between bh (PC-SSFP 84.8 ± 20.3 mL, PC-GRE 82.4 ±19.2 mL) and fb (PC-SSFP 82.6 ± 19.0 mL, PC-GRE 85.2 ± 24.5 mL) PC acquisitions (Fig. 6a), both PC-SSFP (R = 0.98) and PC-GRE (R = 0.99) had strong correlation. However, PC-SSFP showed a smaller intercept (−1.7 vs 16.3) and a slope closer to unity (1.05 vs 0.77) compared to PC-GRE, suggesting that bh PC-SSFP was not compromised by low SNR, and that PC-GRE showed greater disagreement (bh vs fb) due to the lower SNR of bh PC-GRE. Note that fb PC acquisitions were respiratory averaged over all breathing states. Therefore, the slice plane is not identical to bh acquisitions (Figs. 3 and 4).

### 3.5 SNR comparisons

As shown in the histogram (Fig. 6b), the averaged blood SNR across the whole cardiac cycle measured on the magnitude images was significantly higher in PC-SSFP vs PC-GRE (fb: 27.5 ± 8.6 vs 17.7 ± 5.6, *p* < 0.001; bh: 23.7 ± 6.6 vs 10.6 ± 2.9, *p* < 0.001). The SNR improvement was even greater in late diastole (Fig. 6c, fb: 26.1 ± 9.6 vs 12.0 ± 3.6, *p* < 0.001; bh: 22.7 ± 6.9 vs 8.1 ± 2.6, *p* < 0.001). Importantly, single bh PC-SSFP with no averaging demonstrated adequate SNR, which was even higher than three-averaged fb PC-GRE. Considering the similar flow measurement and the fact that VNR is proportional to the SNR, the estimated increase in VNR was also expected to be almost doubled. A simple velocity SD (mm/s) calculation based on the manually drawn ROIs on the static tissues evidenced a similar VNR improvement for PC-SSFP during fb vs PC-GRE (velocity SD 17 ± 12 mm/s vs 42 ± 14 mm/s, 2.5-fold increase, *p* < 0.001).

### 3.6 Comparison with stroke volume by planimetry

**Fig. 7:**
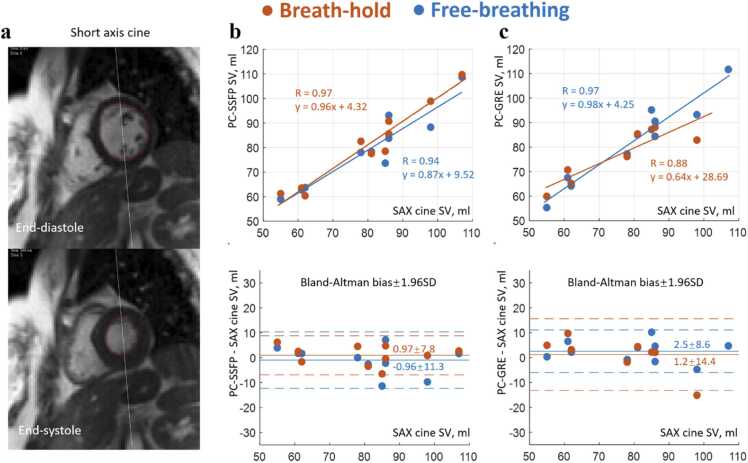
(a) Independent planimetry measurement of stroke volumes using volume difference between end-diastole and end-systole generally showed great agreement with both (b) PC-SSFP and (c) PC-GRE approaches. However, breath-hold PC-GRE had the slope furthest from 1 and the largest bias in the regression results. *PC-SSFP* phase contrast acquisition with balanced steady-state free precession, *PC-GRE* phase contrast gradient recalled echo, *SAX* short-axis, *SV* stroke volume, *SD* standard deviation

Short-axis cine was not acquired in one subject, for technical reasons. Planimetry measurement of stroke volumes in 10 subjects (80.0 ± 16.5 mL) correlated well with PC-SSFP (Fig. 7b) for both fb (R = 0.94; bias ± 1.96 SD = −0.96 ± 11.3 mL) and bh (R = 0.97; 0.97 ± 7.8 mL). Fb (three-averaged) PC-GRE also agreed well (R = 0.97; 2.5 ± 8.6 mL). However, bh PC-GRE had less strong agreement (R = 0.88; 1.2 ± 14.4 mL), with slope furthest from unity and the largest bias in the regression results (SV_PC-GRE_ = 0.64SV_PC-SAX_ + 28.69 mL).

## 4 Discussion

The PC-SSFP method developed here was tested in phantoms and healthy volunteers at 0.55T, showing strong flow quantification agreement with conventional PC-GRE methods, in terms of peak flow, CO, and stroke volumes. The stroke volume measurements agreed with the independent planimetry method using PC-SSFP for both fb and single-average bh acquisitions. Bh PC-GRE with a single average was compromised in CO and stroke volume due to limited SNR, which resulted in less reliable velocity measurement, and aortic contour, especially in late diastolic phases.

Bh PC-GRE resulted in low SNR and VNR, especially during times of low flow (i.e., diastole), since the SNR and thus VNR are dependent on inflow enhancement. This led to noisy velocity maps in some diastolic phases and potentially inaccurate flow measurements. We illustrated the success of our PC-SSFP method to provide superior SNR across the cardiac cycle especially in diastole, suggesting it a preferable approach at 0.55T, and likely also very low fields [23]. Importantly, our one-average PC-SSFP that takes only <20 s demonstrated adequate SNR compared to the standard 2-min fb PC-GRE, permitting improved flow imaging at 0.55T with single bh. However, we noted that PC-SSFP gained a smaller factor of SNR improvement from three averages (only ∼1.2), compared to PC-GRE (∼1.7 vs bh), potentially due to bSSFP’s sensitivity to motion-related artifacts. We also noticed that in two subjects, the flow images from the first two cardiac phases suffered from stronger artifacts, probably caused by disturbances of the steady states.

This study builds on our prior in-plane PC-SSFP approaches at 3T [14], and also the recent 0.55T study by McGrath et al. [12], which found a three-fold increase in VNR with PC-SSFP vs PC-GRE, using inversion of the slice-select gradient for flow-encoding, and a radial self-gating scheme. Our approach differs in that using sign-switched slice-selection gradients [7], [12] for flow-encoding results in linking the slice thickness with the VENC, and this method cannot interleave the flow encodings, as is possible using the current method, because it sets up different steady states. The rapid switching of the large slice-selection gradients can introduce strong eddy currents, artifacts, and possibly a disturbed VENC. Interleaving is generally preferred because there may be less misregistration of the two flow encodings during a bh, or disagreement if the flow values change during the scan. Alternatively, adding a smaller sign-switched bipolar at the end of each TR results in first moment M_z_ nulling only after two TRs [8], so flow encodings cannot be acquired separately. Our sequence used careful zeroth- and first-order nulling at the end of each TR [10], [11] meanwhile maintained a short TR, so that we have the freedom to interleave reference and flow-encoded readouts or acquire them separately without compromising on their respective steady states. Only minor artifacts were observed in a few subjects, due to off-resonance, flow, or motion.

We have previously demonstrated PC-SSFP for in-plane (readout direction) flow at 3T [14], and we anticipate that this is similarly possible at 0.55T. This indicates that it should be feasible for our PC-SSFP method to encode in all directions. At 3T, the additional gradients for PC-SSFP extended the TR, increasing sensitivity to off-resonance, and the gradient design was limited by dB/dt considerations, further extending the TR. But these concerns are mitigated at 0.55T. Additionally, reference-less PC-SSFP [24] may be developed to further shorten the scan time, considering that the phase of bSSFP, with first moment nulled at TE, should be zero or could be approximated by a function of low order, thus may be omitted.

Our proposed PC-SSFP was studied on a 0.55T system with high-performance gradients. By comparison, the commercially available 0.55T system (MAGNETOM Free.Max, Erlangen, Germany) offers reduced gradient amplitude and slew rate (maximum gradient 25 mT/m and slew rate 40 T/m/s), which would lead to a longer TR (∼2.5 ms longer). Translation to a commercial system with lower gradient specifications may compromise the temporal resolution, and thus high-speed inflow velocity measurement. However, TR can be reduced by increasing VENC or lowering spatial resolution, or possibly using a higher bandwidth. Finally, regarding bSSFP off-resonance artifacts, they should still be minimal at 0.55T for TRs as long as 10 ms.

## 5 Limitations

Our PC-SSFP implementation has sampling efficiency penalty by adding extra bipolar gradients to null the first moments, and we did not design our PC-SSFP sequence to null the higher order moments, but no systematic biases in velocity estimation were found in phantom or subject studies. Concomitant field effects are inversely proportional to field strength and may cause extra phase effects in bSSFP imaging, which were not fully investigated. Our method was only studied in healthy subjects for aortic flow. We did not test PC-SSFP in patients and for other applications, such as pulmonary artery flow.

## 6 Conclusion

Our PC-SSFP method can improve the aortic flow image quality at 0.55T CMR, by providing accurate flow measurements and superior SNR across the cardiac cycle, compared to PC-GRE, and by enabling single bh flow measurement.

## Funding

This study was supported by the Intramural Research Program, National Heart Lung and Blood Institute (NHLBI), 10.13039/100000002National Institutes of Health, USA (Z01-HL006257, Z01-HL006213), National Institutes of Health, USA (R01HL144706), and 10.13039/100000968American Heart Association Predoctoral Fellowship (24PRE1177735).

## Author contributions

**Jie Xiang:** Writing – original draft, Visualization, Software, Methodology, Investigation, Formal analysis, Conceptualization. **Rajiv Ramasawmy:** Writing – review and editing, Software, Investigation. **Felicia Seemann:** Writing – review and editing, Investigation. **Dana C. Peters:** Writing – review and editing, Supervision, Conceptualization. **Adrienne E. Campbell-Washburn:** Writing – review and editing, Supervision, Resources, Conceptualization.

## Declaration of competing interests

The authors declare the following financial interests/personal relationships which may be considered as potential competing interests: Adrienne E. Campbell-Washburn reports equipment, drugs, or supplies were provided by Siemens Healthcare and NeoCoil. The other authors declare that they have no known competing financial interests or personal relationships that could have appeared to influence the work reported in this paper.
