# Supplementary material for: Balanced steady-state free precession phase contrast at 0.55T applied to aortic flow
Source: J Cardiovasc Magn Reson. 2024 Sep 13;26(2):101098. doi: 10.1016/j.jocmr.2024.101098 (PMC11638602; doi:10.1016/j.jocmr.2024.101098)
Supplement: Supplemental Material Figs. S1–S3 — Supplementary material [file mmc1.docx]

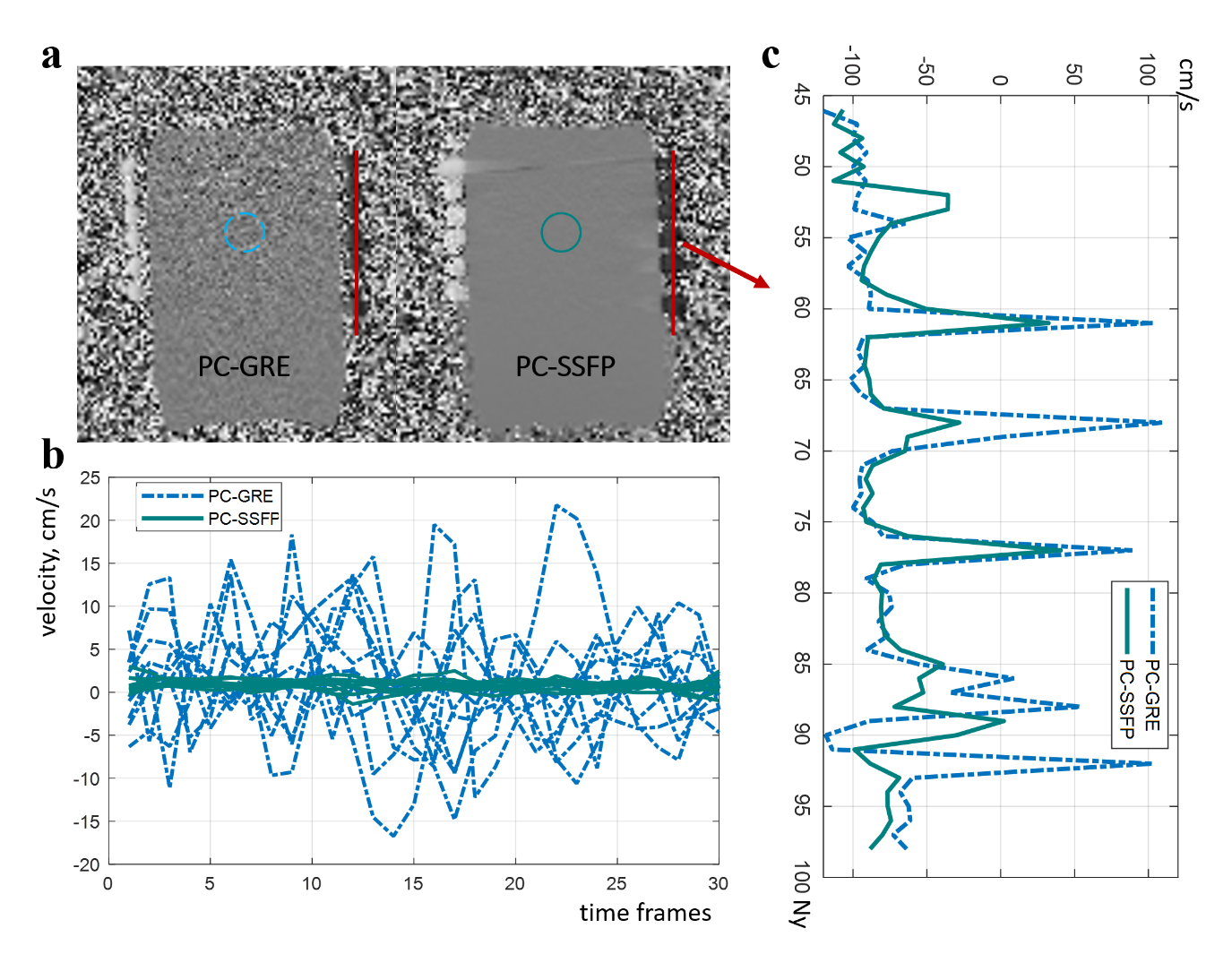


Supplemental Figure S1. Pixelwise comparison between PC-GRE and PC-SSFP. (a) Phase images. (b) Each dashed blue / solid green line refers to one pixel measured by PC-GRE / PC-SSFP within the highlighted circle in (a), respectively. PC-GRE measurements were noisy across different pixels and time frames. (c) Flow profiles across the tube, as highlighted in red line in (a). The measured velocity generally agreed well.


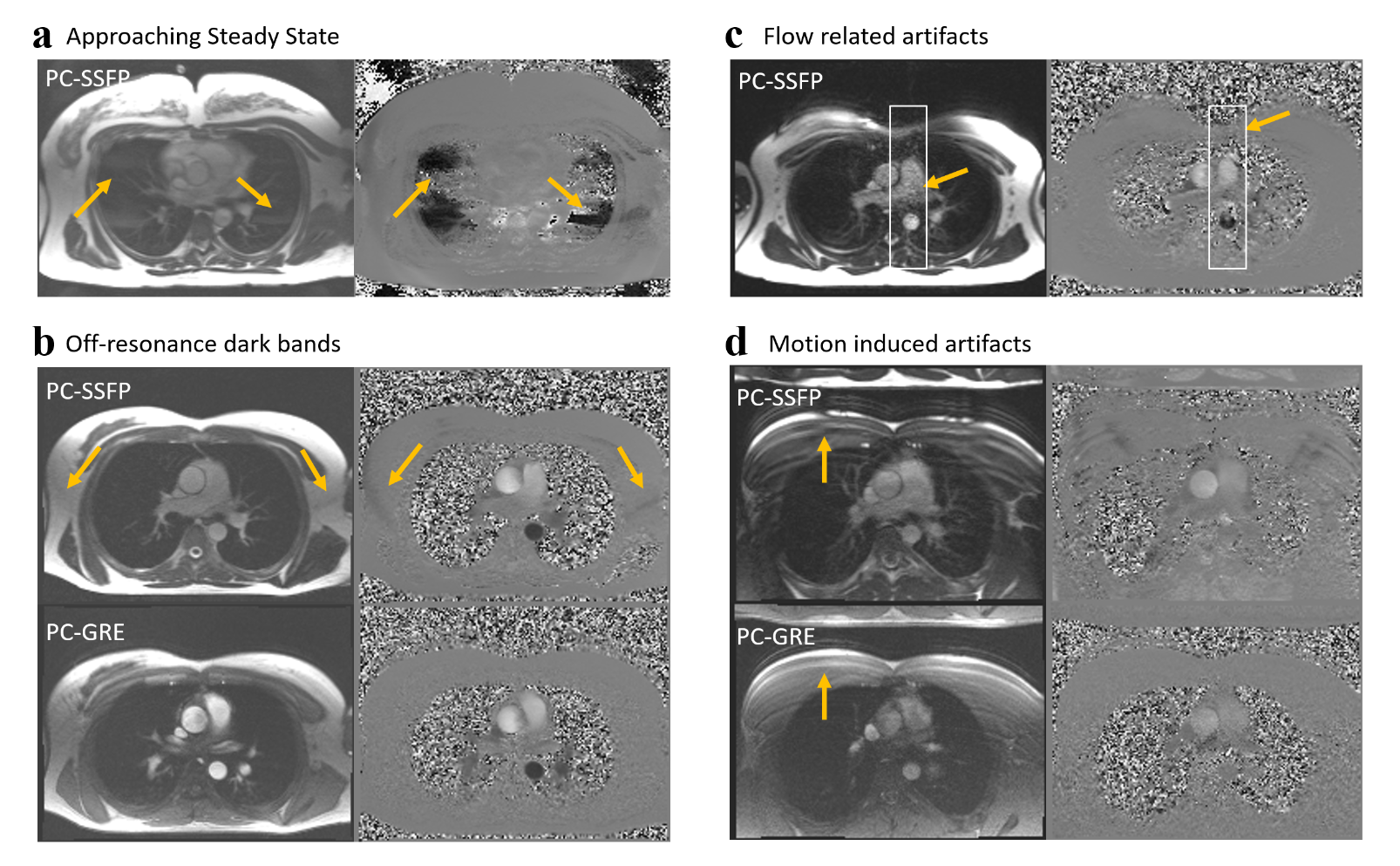


Supplemental Figure S2. Examples of typical artifacts (a) Artifacts in the first several cardiac phases of PC-SSFP, likely approaching the steady state. (b) Off-resonance dark bands that had limited effect on the aortic flow. (c) Minor flow related transient artifacts noticed in only one subject. (d) Strongest motion artifacts, in both PC-GRE and PC-SSFP for the same subject.


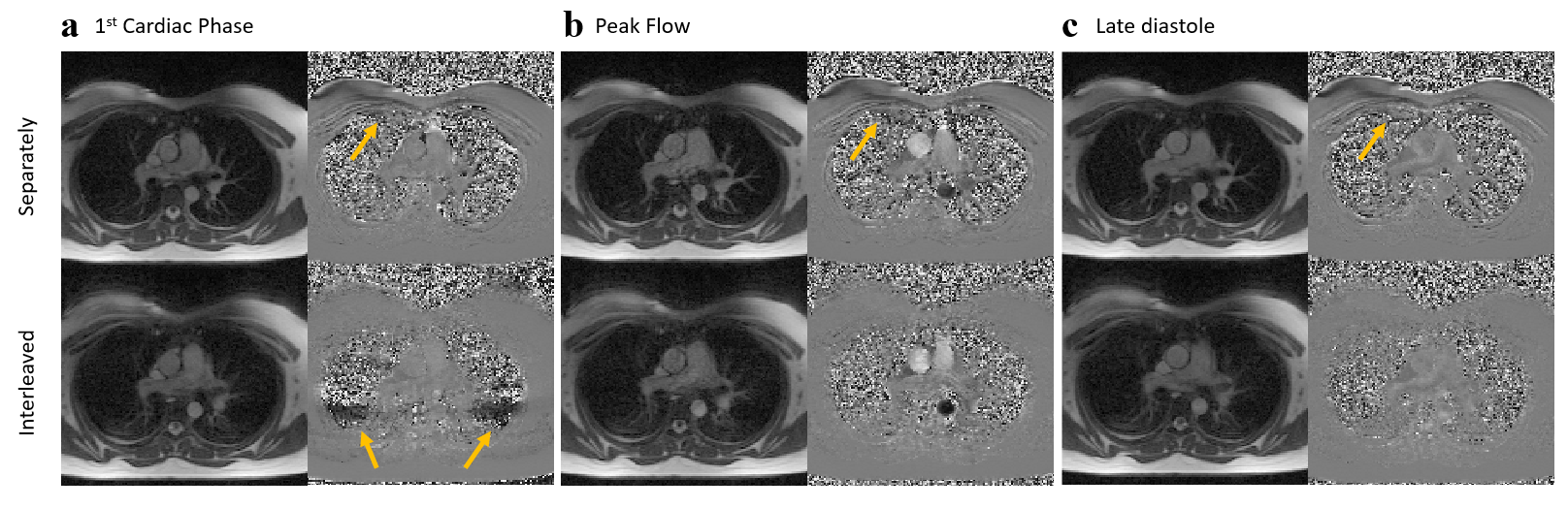


Supplemental Figure S3. Comparison between PC-SSFP acquiring refence/flow-encoded TR in separate (top row) and interleaved (bottom row) approach. Interleaved acquisitions were less sensitive to motion artifacts, sacrificing the image quality in first 1 or 2 frames during reaching steady state. Separate acquisitions did not necessarily provide the same number of reference and velocity encoded TRs using ECG-retro gating, leading to more complexity in reconstruction. Only interleaved PC-SSFP was analyzed and compared to PC-GRE in this work.
